# Clinical variants in *Caenorhabditis elegans* expressing human STXBP1 reveal a novel class of pathogenic variants and classify variants of uncertain significance

**DOI:** 10.1016/j.gimo.2023.100823

**Published:** 2023-06-07

**Authors:** Christopher E. Hopkins, Kathryn McCormick, Trisha Brock, Matthew Wood, Sarah Ruggiero, Kolt Mcbride, Christine Kim, Jennifer A. Lawson, Ingo Helbig, Matthew N. Bainbridge

**Affiliations:** 1 InVivo Biosystems, Eugene, OR; 2 Codified Genomics, LLC, Houston, TX; 3 Division of Neurology, Children’s Hospital of Philadelphia, Philadelphia, PA; 4 The Epilepsy NeuroGenetics Initiative (ENGIN), Children’s Hospital of Philadelphia, Philadelphia, PA; 5 Department of Biomedical and Health Informatics (DBHi), Children’s Hospital of Philadelphia, Philadelphia, PA; 6 University of Pennsylvania, Neuroscience Program, Philadelphia, PA; 7 Rady Children’s Institute for Genomic Medicine, San Diego, CA

**Keywords:** Clinical variant, CRISPR, STXBP1, unc-18, Variant of uncertain significance

## Abstract

**Purpose:**

Modeling disease variants in animals is useful for drug discovery, understanding disease pathology, and classifying variants of uncertain significance (VUS) as pathogenic or benign.

**Methods:**

Using Clustered Regularly Interspaced Short Palindromic Repeats, we performed a Whole-gene Humanized Animal Model procedure to replace the coding sequence of the animal model’s *unc-18* ortholog with the coding sequence for the human *STXBP1* gene. Next, we used Clustered Regularly Interspaced Short Palindromic Repeats to introduce precise point variants in the Whole-gene Humanized Animal Model–humanized *STXBP1* locus from 3 clinical categories (benign, pathogenic, and VUS). Twenty-six phenotypic features extracted from video recordings were used to train machine learning classifiers on 25 pathogenic and 32 benign variants.

**Results:**

Using multiple models, we were able to obtain a diagnostic sensitivity near 0.9. Twenty-three VUS were also interrogated and 8 of 23 (34.8%) were observed to be functionally abnormal. Interestingly, unsupervised clustering identified 2 distinct subsets of known pathogenic variants with distinct phenotypic features; both p.Tyr75Cys and p.Arg406Cys cluster away from other variants and show an increase in swim speed compared with hSTXBP1 worms. This leads to the hypothesis that the mechanism of disease for these 2 variants may differ from most STXBP1-mutated patients and may account for some of the clinical heterogeneity observed in the patient population.

**Conclusion:**

We have demonstrated that automated analysis of a small animal system is an effective, scalable, and fast way to understand functional consequences of variants in *STXBP1* and identify variant-specific intensities of aberrant activity suggesting a genotype-to-phenotype correlation is likely to occur in human clinical variations of *STXBP1*.

## Introduction

Because of the low cost of DNA sequencing as a tool in disease diagnostics, large volumes of variant data are being generated and aggregated in databases such as ClinVar.^1^^,^^2^ Interpretation of these variants by the American College of Medical Genetics guidelines is distributed into 1 of 5 categories: benign (B), likely benign (LB), variants of uncertain significance (VUS), likely pathogenic (LP) and pathogenic (P). VUS are problematic because they are frequently interpreted as nondiagnostic by clinicians^3^—a result that can impede genetic diagnosis for individuals and thereby access to appropriate medical care. Acquiring a diagnosis is critical for obtaining effective therapy, medical reimbursement, and the achievement of a fully informed comprehension for the family or individual affected. Further, designing an effective therapy for a disease requires characterization of the disease mechanism, particularly whether the variant causes a gain or loss of function (GOF, LOF). One of the strategic visions set forth by the National Human Genome Research Institute is to vastly increase clinical relevance predictions, rendering the term “VUS” obsolete by 2030.^4^

**Figure 1:**
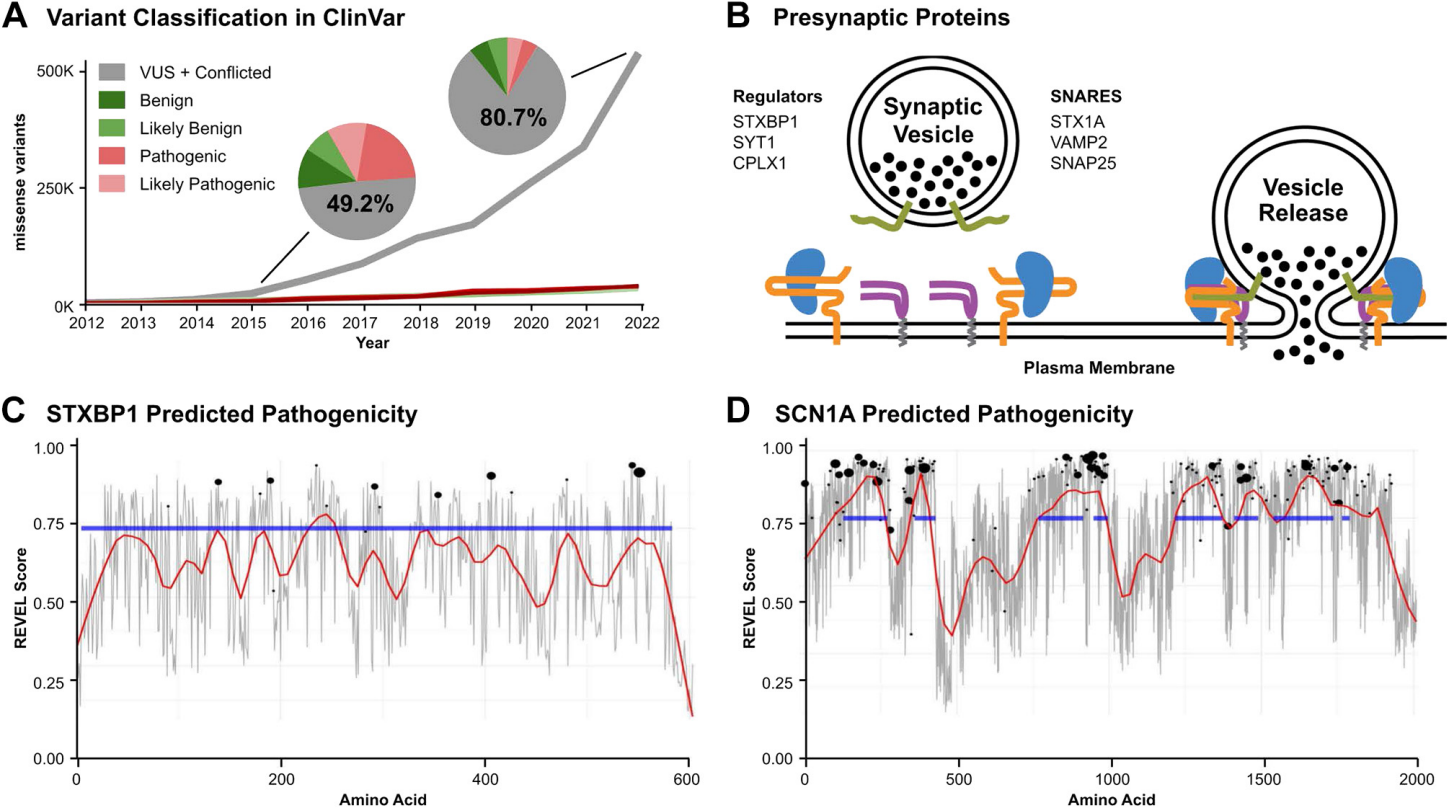
**Growing need for classification in reported variants of STXBP1.** A. Number of missense variants in ClinVar that are VUS (gray), pathogenic (red), likely pathogenic (pink), likely benign (light green), or benign (green) as a function of time. B. The role of STXBP1, the protein of focus in this paper, in coordinating vesicular release. Associated proteins are listed. C. In silico predicted pathogenicity from REVEL, mean score per amino acid (gray), and local regression smoothing (red) across STXBP1, with super family domain (blue), and known pathogenic variants (black dots); dot size is relative to number of pathogenic variants at that locus. D. As in panel C, but for SCN1A. VUS, variant of uncertain significance.

Missense variants are one of the most challenging types of variants to classify accurately.^5^ As of February 2023, there were 660,968 missense variants in the ClinVar database assessed with the 5 classifications of clinical significance (benign [B], likely benign [LB], VUS, likely pathogenic [LP], and pathogenic [P]), with a dominant proportion classified as VUS (72%) and Conflicted (8.7%) (Figure 1A). Prediction of variant pathogenicity can be accomplished in many ways. In silico tools are valuable because they are inexpensive to generate and can be used to precompute every possible variant in the genome. However, even best in class in silico predictions remain only as moderately sensitive tests because of their average receiver-operator curve per gene of only about 80%.^6^ Functional characterization in an animal model is considered strong evidence for variant interpretation. An additional benefit of variant modeling in animal models is the ability to use the animals to screen for new therapeutics.

Mouse models are widely used as human analogs but are challenging to generate and have a high cost per data point. Similar to rodents, the *Caenorhabditis elegans* nematode is a multicellular organism with a variety of differentiated tissues (gut, nerves, muscle, etc.) that interact and give rise to complex behaviors. *C. elegans* is an extremely well-studied model organism—all somatic cells and their lineages are known, behavioral phenotypes are easily quantifiable, and the application of genetic tools and methodologies is advanced for enabling the single-copy, locus-specific insertion of large genetic cargo. In this work, we humanize *C. elegans* by replacing the worm ortholog with the human gene at the native locus, which allows the clinical variant to be analyzed in a gene humanized context. The unique properties of *C. elegans* for fast and affordable gene editing, automated behavioral phenotyping, cellular imaging, and molecular characterization make the nematode a uniquely suitable organism for use in functional studies of clinical variants.

Pathogenic variants in *STXBP1* (NM_003165.3, ENST00000373302) are one of the most common single-gene causes of developmental and epileptic encephalopathy, a neurodevelopmental disorder in which more than 80% of individuals diagnosed have seizure.^7^ Although initially identified in individuals with severe early-onset epilepsy,^8^ the phenotypic spectrum has significantly broadened since, with a large number of individuals presenting with broad neurodevelopmental features.^7^ Infantile spasms, typically starting around the age of 6 months, are one of the most common seizure types in *STXBP1*-related neurodevelopmental disorders. In a recent cohort from a large pediatric health care network,^9^ *STXBP1* was the only genetic etiology significantly associated with infantile spasms and epileptic spasms. Although seizures represent a major issue in infants, developmental issues with a preponderance of communication issues are the main concern in older individuals and adults. The full phenotypic range of *STXBP1*-related disorders is currently unknown, and major questions regarding natural history and features in adulthood remain unanswered.

*STXBP1* encodes a synaptic vesicle protein critically involved in vesicle release. Homozygous Stxbp1 knock-out mice demonstrate completely abolished synaptic function, emphasizing the pivotal role of STXBP1.^10^^,^^11^ STXBP1 has a wide range of functions, including the transport of Syntaxin 1A, a vesicular release protein, from the soma to the synapse via binding at Syntaxin 1A’s Habc subdomain.^12^ STXBP1 also enables Syntaxin to form Soluble N-ethylmaleimide-Sensitive Factor Attachment Protein Receptor at the synaptic terminal and regulate fusion of synaptic vesicles with the plasma membrane^13^, ^14^, ^15^, ^16^, ^17^ (Figure 1B).

Models of STXBP1 dysfunction exist across the spectrum of research animals and cell lines, including yeast, flies, worms, mice, HEK293 cell culture, and differentiated fibroblasts. However, 2 animal models, mouse and *C. elegans*, have emerged as the main organisms of research for the study of variant function, as published by several groups.^18^, ^19^, ^20^ Using these organisms, researchers have recently been able to provide evidence for differing disease mechanisms, such as haploinsufficiency, in which a protein truncation or nonsense variant results in a loss of translated functional protein, and dominant negative function, in which missense variants result in misfolded proteins that aggregate with and disrupt the function of wild-type copies.^20^ Only recently, protein stabilization strategies have been identified and are currently being assessed in clinical trials (NCT04937062).^21^

Therapeutic discovery for *STXBP1* disorders has been undertaken using structure-based in silico screening of 255,780 compounds paired with characterization of 17 variants in HEK293 cell culture and 3 variants in *C. elegans* model.^21^ This approach was successful in identifying 3 commercially available compounds that were capable of rescuing STXBP1 protein levels, as well as functional spontaneous and evoked neurotransmission in 2 disease associated variants, p.Glu544Asp and p.Arg406His. Intriguingly, of the 3 compounds explored on 2 variants, each variant had a unique compound that was most capable of restoring protein stability and function, indicating that variant-specific therapies may be an important approach to STXBP1 disease.

Nearly half of *STXBP1* ClinVar variants are VUS. Using in silico prediction tools, such as REVEL^22^ and BayesDel,^23^ is challenging for *STXBP1* variant determination because the protein does not have multiple distinct functional domains (Figure 1C). In contrast, with another important epilepsy gene (eg, *SCN1A)*, the same ensemble predictors are able to show regions of high predicted pathogenicity and that these “hot spots” overlay onto superfamily domain structure (Figure 1D). Because the predicted pathogenicity for variants in *STXBP1* is relatively uniform across the protein, there are no specific regions that can be categorically regarded as LB/P. This analysis suggests that functional analysis of variants in an animal model will be necessary to resolve the majority of VUS in *STXBP1*.

## Materials and Methods

### Creation of humanized wild-type animals

The coding sequence for the most abundantly expressed isoform of *STXBP1* (isoform a; NM_003165.3) was extracted from the UniProt database (https://www.uniprot.org/). The sequence was codon-optimized for transgene expression in *C. elegans*. Three synthetic introns were introduced, and the sequence was further optimized for enabling splicing specific only to the introduced introns. The sequence was introduced into the native *unc-18* locus using Clustered Regularly Interspaced Short Palindromic Repeats (CRISPR) gene editing. Two single guide RNA (sgRNA) sites in *unc-18* were selected, 1 in the 5′UTR of *unc-18* and the other in the last exon of *unc-18*. A plasmid was designed to provide donor homology sequences that flank the outside edges of the two cut sites. Within the plasmid sequence and between the 2 donor homology sequences, the codon-optimized *STXBP1* sequence was introduced using standard molecular cloning techniques. Care was taken to design for elimination of the sgRNA sites to avoid recutting of the edited locus. The plasmid also contained a Hygromycin resistance cassette for selection.^24^ The plasmid along with Cas9 and sgRNAs was injected into the hermaphrodite gonad to elicit transgene insertion.^25^ Animals were harvested by Hygromycin B selection for transgene insertion and homozygous animals were identified.^26^ Verification of the desired edit was confirmed by polymerase chain-reaction (PCR) and was followed by DNA sequencing. After confirmation of the desired edit, an additional round of genome editing was performed to remove the antibiotic selection sequence and restore the native 3′UTR sequence. A second round of PCR and DNA sequencing was performed to confirm that the desired sequence composition of the humanized *STXBP1* transgenic worm was obtained (the “*hSTXBP1*” strain). After strains were genotypically confirmed, we quantified mRNA expression as a validation that the variants introduced into the coding sequence were being transcribed.

### Creation of variant animals

For each variant, a set of sgRNA were selected to flank the locus of interest. A donor homology oligonucleotide was designed to have at least 35 base pairs of homology on the outside ends of the cut sites. In the interval between the cut sites, the DNA was recoded with a new sequence, containing both the desired amino acid change and silent variants that block recutting. The donor homology oligonucleotide was co-injected into the hermaphrodite gonad with the appropriate sgRNAs and Cas9 and dpy-10 co-CRISPR reagents. Animals were harvested for transgene insertion and homozygous animals were harvested. Verification of the desired edit was confirmed by PCR followed by DNA sequencing, and transgenic lines were also confirmed to be wild type at the dpy-10 co-CRISPR locus. We found no significant differences in mRNA expression between variants and control sequences. We also performed an in silico assessment for any unintentionally introduced splice variants and confirmed that all flagged variant lines spliced as expected via sequencing, suggesting that splice variants are not occurring in vivo. This inability to detect splice variation with the synthetic introns chosen is consistent with the prior humanization constructs where a majority of the transgene sequence yielded a functional protein and lesser fraction (∼20%) yield a coding sequence that missplice into a nonfunctional protein that cannot rescue the loss of the endogenous coding sequence.^27^

### Deep phenotyping data acquisition

Ten adult animals from bleach synchronized populations were transferred to clean Nematode Growth Medium plates seeded with HB101 bacteria. A copper ring was melted into the agar to keep the worms from escaping the field of view. Worms were allowed to acclimate for 10 minutes on the plates after which videos were recorded for 10 minutes using the WormLab platform (MBF Bioscience). For each variant 3 independent biological replicate populations were assayed. The WormTracker software (MBF Bioscience, version 2020.1.1) was used to track each worm’s movement throughout the 10-minute recording time. Tracks were repaired to account for collisions between worms and with the copper ring. Nonworm signals were removed. The WormTracker software was then used to analyze and export the worm movement behavior and morphology features.

### Single phenotype analysis

In situations where a single phenotype is analyzed alone, the phenotype was compared using Student’s *t* test.

### K-means clustering

Data for known P/B genotypes were scaled and centered using the R-package Caret v6.0.94 (Max Kuhn, 2020). K-means was then performed with R v4.3 (R Core Team, 2018) and the k-means function (stats package, v4.3). To determine the number of clusters we utilized the “elbow method.”^28^ To visualize k-means clusters, we reduced the data down to 2 dimensions using principal component analysis (stats package v4.3, princomp() with cor = True). The first 2 principal components for each genotype were plotted using ggplot2^29^ (v3.4.2) using the cluster assignments from the k-means analysis. VUS were then superimposed onto the existing clusters.

### Machine learning (ML) and classifiers

We trained our classifiers on both unaggregated (raw) data and data aggregated by genotype, by taking the mean across worms of the same genotype for each feature. There was no significant difference in classification metrics. The Support Vector Machine (SVM) was implemented using SciKit Learn version 0.24.1 in Python 2. Five-fold cross validation on the training set was performed to select the best performing learning algorithm and hyperparameters. An SVM with Radial Basis Function kernel and gamma = .137, C = 1 was selected. The features were normalized before training began. Our Random Forest (RF) model was produced using R and randomForest^30^ v4.7.11. We built the RF using all available input features. To determine values for mtry and ntree we built RFs across a range of possible values and plotted the out-of-bag errors. Altering these parameters did not significantly affect the performance of the RF. To determine performance we generated the fraction of out-of-bag pathogenic votes for each training genotype of our training genotypes (mtry = 15 and ntree = 500). This vote fraction shows classification agreement among trees and can be used as a confidence score.

### ClinVar analysis

The ClinVar database was used to examine the clinical significance of missense variants as a function of time. ClinVar was queried with creation dates for variants on an yearly calendar (ie, “2010/01/01 [Creation Date] : 2010/12/31 [Creation Date]”). The results were then restricted to missense variants. The number of variants in each Clinical Significance category were then recorded from the filter table at left and plotted as a function of ending year.

## Results

### Insertion of human *STXBP1* transgene as gene replacement of the *C. elegans unc-18* gene results in the rescue of LOF activity

Clinical variant modeling can be performed as either amino acid change in the ortholog locus (native) or as an amino acid change in the whole-gene humanized locus (“gene swap”). For *STXBP1*, modeling variant function by making an amino acid change in the *unc-18* locus is problematic. Because sequence identity between *STXBP1* and *unc-18* is only 59%, only a fraction of VUS can be modeled with the native locus approach. To circumvent this limitation, the gene-swap method was used to allow the entire gene locus to be humanized by replacing the endogenous *unc-18* coding sequence with a human transgene sequence coding for the human *STXBP1* wild-type gene (*hSTXBP1*).

**Figure 2:**
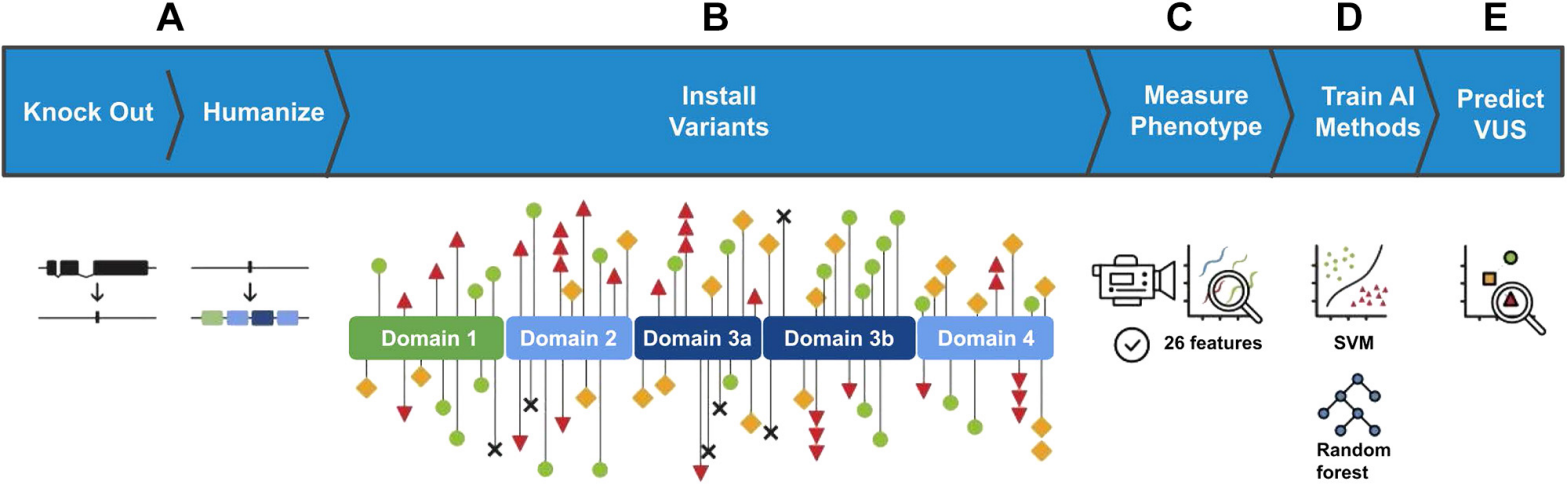
**Schematic representation of the experiments performed.** A. The native homolog of *STXBP1*, *unc-18*, was removed from the genome in a full deletion knockout. Subsequently, a codon optimized coding sequence encoding human *STXBP1* is inserted into the same genomic location. B. Individual human variants were created in the *STXBP1*-expressing animals. The functional domains of *STXBP1* as determined via crystallography are depicted, and the location of individual variants are marked with shapes. Red triangles represent pathogenic missense variants in our training data set, black X’s represent pathogenic truncating variants, green circles represent benign variants, yellow squares represent VUS. C. The generated strains were automatically assessed for 26 phenotypic features characterizing the animals’ movement and morphology. D. Two machine learning models, random forest and SVMs were trained on the resulting data set. E. Models were used to sort VUS into functionally normal and abnormal groups, representing functional predictors of pathogenicity. SVM, support vector machine; VUS, variant of uncertain significance.

The *hSTXBP1* sequence was inserted at the orthologous locus using CRISPR-based gene editing techniques (Figure 2A and B). The result was a genomic-integrated transgene that is regulated by endogenous *unc-18* regulatory sequences. Locomotion analysis showed a partial rescue of phenotype compared to wild-type levels. This observation of partial rescue contrast with prior work of Zhu et al^19^ wherein complete rescue of function was observed. For the remainder of this work, we treat the partial rescue as our new “baseline” and all variant effects to worm phenotype will be measured against the *hSTXBP1* worm. Possible reasons for this discrepancy of outcomes are the choice of coding sequence or the likeliness of higher expression when comparing transgene expression from extrachromosomal arrays with single-copy germline-encoded expression (the work herein).

### Creation and deep phenotyping of *C. elegans* encoding human clinical variants

We used CRISPR-based gene editing to introduce clinically relevant variants into the *hSTXBP1*-*wt* locus. A set of 81 clinical single nucleotide human population variants listed in ClinVar were selected to be modeled in consultation with clinicians and patient advocacy groups, including 25 benign, 32 pathogenic, and 23 VUS (Supplemental Table 1). We classified the variants for input into our ML models according to standardized criteria. The benign variants were those that had a B/LB designation in the ClinVar database, or those that had a high frequency as reported in the gnomAD unaffected control population database (3 observations or a frequency of 1.19e-5). This minor allele frequency threshold was picked because it is on the same order of magnitude of the incidence of STXBP1 driven disease.^31^ The pathogenic variants were those that had pathogenic and likely pathogenic annotations in the ClinVar database as of January 2021. To ensure LOF phenotypes would be present, 6 of the pathogenic variants were selected from protein truncation variants that disrupt full-length expression at various positions along the primary amino acid sequence (p.Arg122Ter, p.Arg367Ter, p.Arg388Ter, p.Tyr140Ter, p.Glu302Ter, and p.Lys308Ter). The variants that met the criteria for pathogenic and benign were used as a training set. The VUS were selected from ClinVar and in consultation with various clinical groups. For control strains the *N2* (wild type), *unc-18* (*COP1385 [knu395]*, full gene knockout [KO]), and *hSTXBP1-wt* (*COP1781 [knu692]*, human *STXBP1* as substitution for *unc-18* coding sequence). The resultant strains were phenotyped using a semiautomated system in which locomotion and morphology of individual animals were video recorded and quantified via software algorithm (Figure 2C-E). In total, 2956 animals were phenotyped in this study (av 35.2 +/− 9.5 animals per genotype) using computer analyzed video recordings of animal movement on agar.

### Data exploration and feature engineering

**Figure 3:**
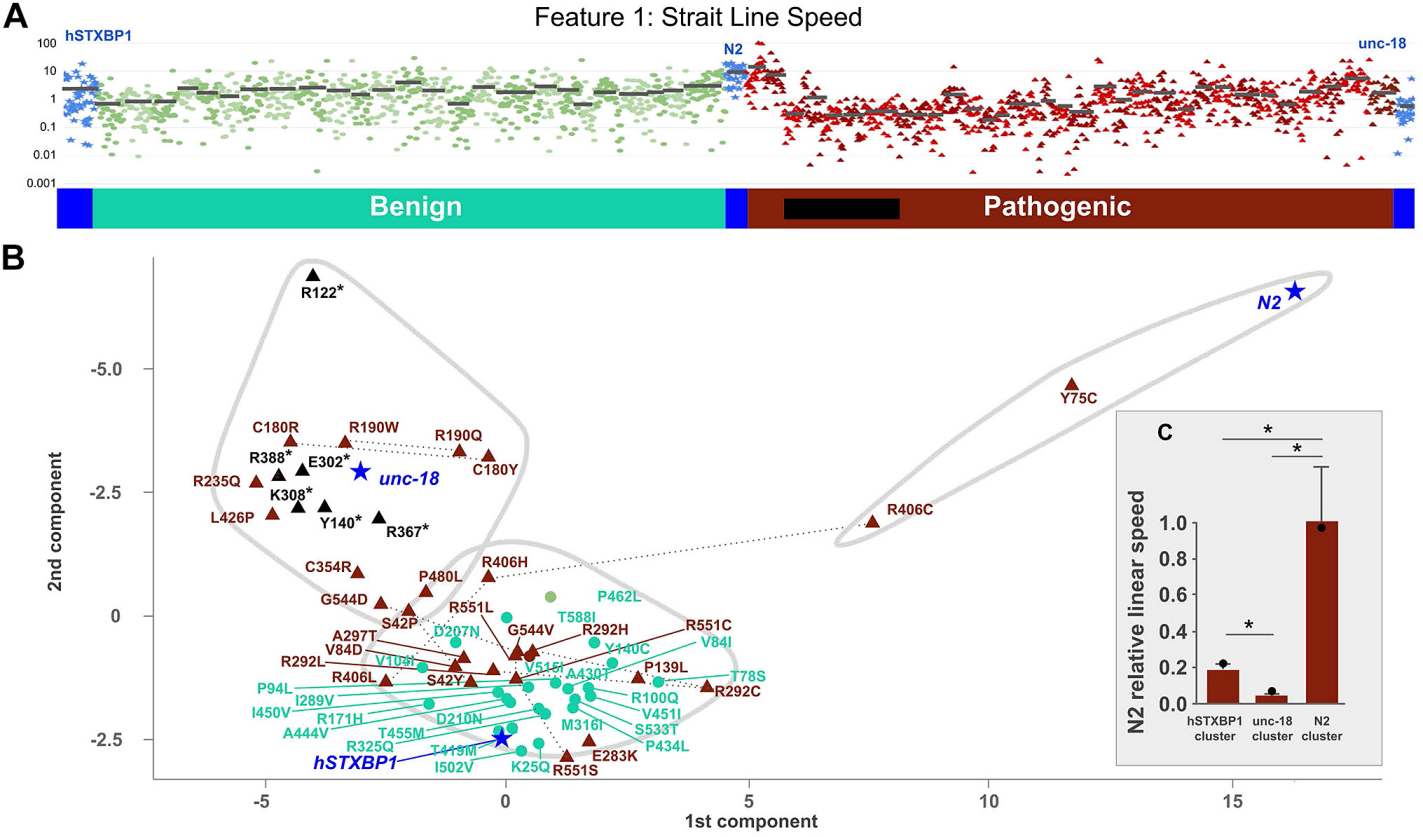
**Example data and unsupervised cluster analysis**. A. Straight Line Speed measured across 2034 worms from 60 genotypes: 25 benign (cyan) and 32 pathogenic variants (brown) plus controls (blue: unc-18 full deletion null, hSTXBP1 whole-gene humanized, and N2 wild-type) (average strain value marked by black bar). B. Pathogenic missense (brown triangles), pathogenic truncating (black triangles), benign (cyan circles), and control (blue stars) variants were clustered using k-means algorithm and plotted in 2 dimensions (principal components 1 and 2), which shows 3 distinct clusters (gray-bound regions). C. Inset bar graph shows linear speed for each pathogenic cluster, with standard error (whiskers) and speed of the control sample in each cluster (black circle). Asterisk indicates *P* value < .05 (*t* test). R:Arg; N:Asn; D:Asp; C:Cys; E:Glu; Q:Gln; G:Gly; H:His; I:Ile; L:Leu; K:Lys; M:Met; F:Phe; P:Pro; S:Ser; T:Thr; W:Trp; Y:Tyr; V:Val.

Primary data analysis and exploration indicated that no single feature could be used as a reliable indicator of functionally abnormal protein activity to delineate pathogenic and benign variants. For instance, the Straight Line Speed feature showed significant overlap on an individual worm basis (Figure 3A). We conducted principal component analysis and determined that the first 2 principal components only accounted for 64% of the variance in the data, indicating that there was very little redundancy in the data (data not shown). This analysis also suggested that significant information would be lost in dimensional reduction. Thus, we performed k-means clustering using all 26 features as inputs and subsets of features were identified with possible LOF and GOF effects (Figure 3B). We determined the optimal lower bound for K was 3 (see Methods) and plotted the resulting clusters in 2 dimensions for visualization. Interestingly, each cluster was found to contain 1 of each control: *unc-18* and truncating variants, a mix of pathogenic and benign variants with *hSTXBP1*, and 2 variants (p.Tyr75Cys and p.Arg406Cys) clustered with the wild-type N2 worm. Examining linear swim speed (Figure 3C) showed strong discrimination between each cluster, with the N2 cluster showing the highest swim speed, *unc-18* showing the lowest, and *hSTXBP1* showing an intermediate phenotype.

### Training of ML algorithms

**Figure 4:**
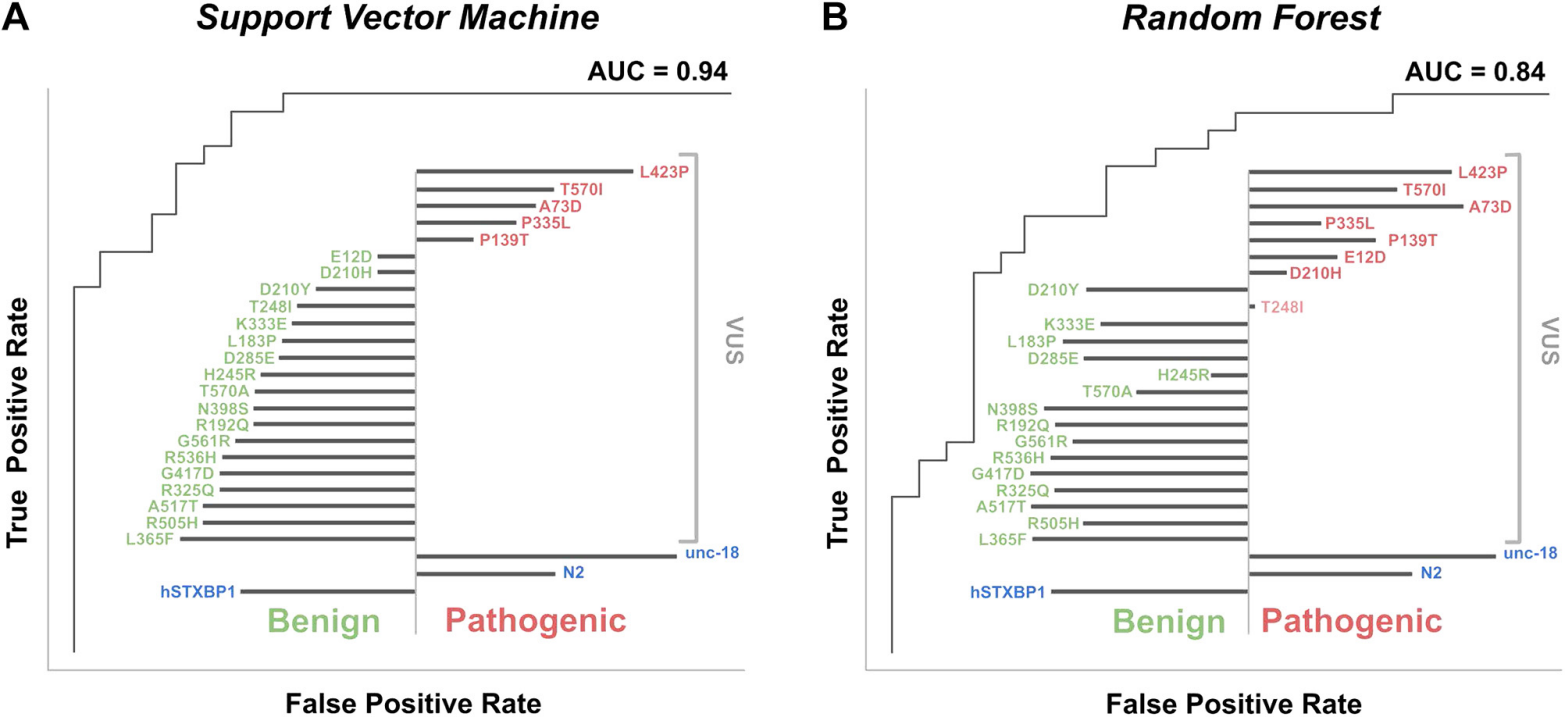
**Model evaluation and functional predictions using supervised machine learning algorithms.** VUS predicted pathogenic (red text) and benign (green text) are shown; the length of black bar (left for benign, right pathogenic) indicates strength of classification. A. A support vector machine classification, of known benign and pathogenic variants, achieved an AUC of 0.94 and classified 5 of 23 VUS as pathogenic. B. A random forest classification achieved an AUC of 0.84 and classified 8 of 23 VUS as pathogenic. R:Arg; N:Asn; D:Asp; C:Cys; E:Glu; Q:Gln; G:Gly; H:His; I:Ile; L:Leu; K:Lys; M:Met; F:Phe; P:Pro; S:Ser; T:Thr; W:Trp; Y:Tyr; V:Val. AUC, area under curve.

In order to generate a phenotype-based decision boundary for VUS, we undertook training of 2 ML classifiers with the established pathogenic and benign variants (Supplemental Table 1). We used 2 orthogonal classifier algorithms: RF, which generates a classification status based on the majority call vote of a series of decision trees, and SVMs, which uses training data to generate a hyperplane decision boundary that can be used to classify new inputs. We evaluated the performance of the classifiers by examining the confusion matrices, receiver operator characteristic plots, and precision recall plots. Both classifiers performed well. The RF had an area under the receiver-operator curve of 0.84 and an area under precision-recall curve of 0.87, whereas the SVM had an area under the receiver-operator curve of 0.94 and an area under precision-recall curve of 0.96 (Figure 4A and B). The 2 classifiers were in good agreement, differing on their classification on only 4 of the 57 variants in the training set. However, both classifiers misclassified the pathogenic variants p.Arg551Cys, p.Arg551Ser, p.Arg551Leu, p.Glu283Lys, p.Gly544Val, p.Arg292His, p.Arg292Leu, and p.Ser42Tyr as benign. They also both misclassified the benign variants p.Asp207Asn, p.Val104Ile, and p.Thr588Ile as pathogenic variants. The truncation variants were consistently classified correctly and were among the most pathogenic according to the distance from the decision boundary (SVM) or percentage of decision tree votes (RF).

### Evaluation of VUS

Of the 23 VUS evaluated by the 2 models, the SVM and RF both classified 5 variants as pathogenic: p.Leu423Pro, p.Thr570Ile, p.Ala73Asp, p.Pro335Leu, and p.Pro139Thr (Figure 4). Subsequent to this analysis, the latest version of ClinVar (Dec 2022) has at least 3 assertions and lists p.His245Arg and p.Leu423Pro as P/LP and p.Pro335Leu as conflicting, see Supplemental Table 1 for detail of ClinVar assessment. Our model disagrees with ClinVar’s assertion for p.His245Arg but agrees with p.Leu423Pro. Both models assert that p.Pro335Leu is pathogenic. The classifiers disagreed on 3 variants (p.Asp210His, p.Glu12Asp, and p.Thr248Ile) with the RF classifier judging them as pathogenic, whereas the SVM classifier judged them as benign. All other VUS were classified as benign by both methodologies.

## Discussion

This work demonstrates that gene-humanized animals expressing *STXBP1* can be used as a line of functional evidence in determining variant pathogenicity and identify distinct phenotypic subclusters of pathogenic variants. 83 genotypes were analyzed in various classes of variants (25 benign, 32 pathogenic, 23 VUS, and 3 Controls [KO, hSTXBP1, and N2]). The variants created and functionally assessed in this paper account for a significant proportion of variants seen in individuals with *STXBP1* related disorders, including most recurrent variants in the *STXBP1* gene. In this work the 32 pathogenic variants screened represents approximately 25% of all known pathogenic variants in STXBP1, reflecting the scalability of our assay system.

Early-Infantile Epileptic Encephalopathy 4 is an autosomal dominant disorder, which manifests in individuals heterozygous for disease causing variants within *STXBP1*.^32^ The animal models herein are phenotyped in their homozygous state. As a result, the biallelic effect of the clinical variants is being modeled, and mechanisms such as haploinsufficiency and dominant negative function cannot be resolved. In our assay, both classifiers struggled with the known mutational hotspots Arg551 and Arg292, misclassifying 3 of 3 and 2 of 3 variants, respectively; however, the classifiers correctly identified all variants at the hotspot Arg406. Further investigation into the mechanism of pathogenicity for variants involving these positions is needed. It is possible that our biallelic modeling results in an inability to detect dominant negative functions.

Appreciating the value of these assays in determining the pathogenicity of VUS is complex. According to previously determined criteria,^33^ our assay reaches the threshold of pathogenic support PS3_moderate because it assays >10 control (positive [*n* = 25] and negative [*n* = 32]) variants selected from well curated databases with multiple biological replicates (*n* = 3). This approach is complicated by the fact that we measure multiple phenotypes at once and evaluate the pathogenicity using ML approaches as opposed to straight forward statistical tests (eg, *t* test). However, we can take advantage of the score of the strength of the prediction to formally calculate the probability of the assay being correct: for example, our over-all SVM prediction of pathogenicity (score >0) is 88% but rises to 95% with a score >0.2 and 100% with a score >0.3. Thus, this approach may also be considered PS3_strong if the ML score is high enough.

Despite the limitations, the findings presented herein represent the development of a fast and efficient platform for functional testing of clinically observed variants in a disease-associated gene that can be applied in a whole animal context. Approaches in live animals may present an important alternative to deep mutational scanning in cells. Although extremely powerful and efficient, deep mutational scanning requires that the gene being analyzed results in a lethal phenotype, which may be either inherent or in an engineered genetic context. Additionally, because genes have different functions in different tissues, mutational analysis in a single cell type may not be representative of overall organismal results. In vivo mutational modeling, as performed here, has the additional benefit of enabling therapeutic discovery on behavioral phenotypes and allowing simultaneous discovery of mechanisms (eg, GOF and LOF) that can be tied to molecular readouts (eg, mRNA and protein expression). Performing the equivalent level of clinical variant modeling with germline integration of single nucleotide polymorphisms in the mouse model would require a much longer timeline before any phenotypic evaluation could be performed. Although disease modeling and mechanism research is advancing quickly in the zebrafish, currently the extreme difficulty of generating point variants via homology directed repair is rate limiting. Performing CRISPR based homology directed repair editing in *C. elegans* is routine and can be performed on timelines relatively close to cell culture. To our knowledge, such an extensive variant analysis in a disease-associated gene has not been performed in any other model organism. The results of this study could inform future study designs in, for example, iPSCs, which would provide an important validation.

This VUS analysis platform could be applied to other genes in which functional assessment is required, but deep mutational approaches are not available. We suggest that candidate genes for the approach taken herein have several qualities: (1) homology between the human and *C. elegans* genes is >50%, (2) the human disease gene has 2 or fewer high-homology orthologs in *C. elegans*, and (3) the overall size of the human coding sequence is <5kb. A preliminary analysis of the allelic variants with genotype-phenotype relationships cataloged in the OMIM database indicated that 2058 of 4689 (44.5%) would meet these criteria. As a result, many human genes are likely to be amenable to the same gene humanization technique applied to *STXBP1* and variant pathogenicity can be tested in a whole animal format.

Discrimination between LOF and GOF variants may be key in appropriately choosing therapeutics for individuals with disease-causing variants. Finding both GOF and LOF variants is common in dosage-sensitive genes including many genetic etiologies implicated in the epilepsies, such as *SCN2A* or *SCN8A*.^34^^,^^35^ In KCNQ2-associated diseases, the use of antiepileptic drugs effective for LOF variants of *KCNQ2* is associated with poor outcomes in patients with a GOF variant.^36^ Differentiating between GOF and LOF variants can give key insights into the prognosis expected for an individual. Interestingly, though most pathogenic and truncating variants occupied a similar phenotypic feature space as the native *unc-18* KO in our assay, 2 pathogenic variants (p.Tyr75Cys and p.Arg406Cys) seem to act more similar to wild-type worms than humanized worms, specifically in linear swim speed (Figure 3). This suggests that they have increased synaptic release compared with the unmutated humanized worm and the variants represent a GOF phenotype that can be discerned by this in vivo assay. Interestingly, other variants at Arg406 do not show increased movement speed and may indicate an entirely different mechanism of pathogenesis at this locus for this variant. Indeed, the mechanism of action is difficult to determine: protein domains in *STXBP1* are all large and nonspecific to these variants. Although both variants result in producing a cysteine residue, which can form disulfide bonds, only Arg406 is proximal to another cystine (Cys366, ∼7.5Å). Although cystines can form other post-translational modifications, many of these are transient^37^ and may not account for the phenotype. Other studies show increased stability for STXBP1 proteins containing the p.Arg406Cys variant, which may account for the apparently GOF phenotype;^38^ however, independent functional understanding on the p.Tyr75Cys GOF phenotype is not available. Although the mechanism of disease is unclear, this assay indicates that special attention should be paid to these variants.

There is growing recognition of GOF variants in STXBP1,^18^ though this is the first report of known heterozygous pathogenic variants that seemingly cause a GOF phenotype. Interestingly, when we overlay our k-means clustering results with VUS, p.Pro335Leu co-clusters with our putative GOF group of variants hinting that there may be many more GOF variants in this gene. Understanding disease mechanism is extremely important when evaluating therapeutics, typically with more options available for GOF than LOF variants.^39^, ^40^, ^41^ Importantly, these variants are scored as pathogenic by REVEL (>0.9) but are not discernible from other (LOF) pathogenic variants illustrating the importance of modeling variants in a biologically relevant manner.

Despite the accuracy observed with the SVM analysis, the humanized worm did not achieve a complete rescue of the *unc-18* KO phenotype when the animals were video monitored for movement and morphology on solid growth media (Figure 3). STXBP1 is a presynaptic protein whose primary role is to provide coordinated release of neurotransmitters at the synapse. STXBP1 directly binds syntaxin and influences syntaxin's binding interaction with the 2 other key proteins necessary for promoting vesicle fusion, synaptobrevin and SNAP25. The lack of complete rescue with the human transgene may be due to imperfect fit between STXBP1 and the *C. elegans* subunits of the SNARE complex. Because the common ancestor of *C. elegans* and humans was 500 million years ago, the presynaptic proteins have coevolved as their sequence drifted over time. Future work to introduce multiple human proteins into the *C. elegans* synapse may increase the predictive power of this animal model system.

### Conclusions

We describe a fully humanized *C. elegans* model for rapid modeling of the pathogenic effects of variants in *STXBP1*, a common cause of genetic epilepsy. We demonstrate that our genetic models can be used within automated phenotypic analysis and ML workflows to distinguish disease-associated pathogenic variants from benign variants, thereby providing a scalable assay to understand variant pathogenicity and VUS resolution. We used the disease modeling platform to functionally characterize 23 VUS and present evidence that 8 of the VUS (35%) are functionally abnormal. Although further assessment is needed, including testing on other known GOF variants, this assay appears to be able to distinguish between GOF and LOF variants, which is critical for therapeutic development.

## Data Availability

All data are made available in the supplemental information.
